# A novel use for suture button suspension: reconstruction of the dorsal ulnar ligament to treat thumb metacarpal dislocation

**DOI:** 10.3109/23320885.2014.997823

**Published:** 2015-01-06

**Authors:** Ajul Shah, Garry Martin, James Grant Thomson

**Affiliations:** ^a^ Section of Plastic and Reconstructive Surgery, Yale University School of Medicine, New Haven, CT, USA

**Keywords:** Hand, plastic surgery, suture button, mini tight rope, dorsal ulnar ligament, thumb CMC, CMC joint

## Abstract

There are numerous treatment algorithms that have been developed to treat thumb carpometacarpal (CMC) arthritis. A newer treatment option for these patients is CMC stabilization using suture button suspensionplasty. The authors of this case report have extensive experience with the suture-button suspensionplasty using the Mini TightRope CMC technique (Arthrex). We present a novel usage of the suture-button suspensionplasty to reconstruct the dorsal ulnar ligament (in contrast to the usual reconstruction of the volar beak ligament) to treat a patient with persistent thumb metacarpal dislocation at the CMC joint. Two separate patients are presented. One patient demonstrates volar beak ligament instability, and the other demonstrates dorsal ulnar ligament instability. Both patients’ demographics and operative indications are described. The operative technique for the novel usage of the suture-button suspensionplasty is described. Operative results of the dorsal ulnar ligament reconstruction are reviewed. After suture-button suspension of the thumb metacarpal to the trapezium, the dorsal ulnar ligament has been reconstructed. The patient demonstrated stability of the thumb CMC joint without dorsal or radial dislocation. The authors of this case report present a novel usage of the suture-button suspensionplasty to treat a patient with proximal thumb metacarpal dislocation at the trapezial-metacarpal interface. This method, in contrast to the referenced method of volar beak ligament reconstruction, allows reconstruction of the dorsal ulnar ligament. This allows stabilization of the joint by preventing dorsal and radial dislocation of the metacarpal.

## Introduction

Thumb carpometacarpal joint arthritis remains a difficult entity for hand surgeons to treat. The first metacarapal and the trapezium articulate to form a unique joint, often termed a ‘saddle joint’. The axes of the opposing saddles are perpendicular to each other, such that the distal saddle faces proximal and the proximal saddle is rotated 90° in relation to the distal upside-down saddle. The anatomy of the joint allows thumb motion in extension, flexion, adduction, and abduction [1]. The ligamentous anatomy of the thumb carpometacarpal (CMC) joint is important for stability. Several ligaments play a major role in the stabilization of the thumb CMC joint. The palmar ligament, also known as the oblique or beak ligament, acts as a static restraint by virtue of its intracapsular location. It originates from the palmar tubercle of the trapezium and inserts into the articular margin of the ulnar side of the metacarpal base [2].

There are numerous treatment algorithms that have been developed to treat thumb CMC arthritis. Non-surgical treatment options include hand therapy (stretching and strengthening exercises), splinting, and injection. Operative treatment options include, but are not limited to, metacarpal extension osteotomy [3, 4], thumb CMC arthrodesis [5], implant arthroplasty [6, 7], and resection arthroplasty (partial or full trapeziectomy) with or without ligamentous reconstruction and tendon interposition [8, 9, 10, 11, 12, 13], all with varying results. A newer treatment option for these patients is CMC stabilization using suture button suspensionplasty. This technique overcomes the limitations of K-wire fixation by using a suture-button implant to resist subsidence [14]. The suture button device consists of braided polyester sutures (Fiberwire, Arthrex, Naples, FL) looped between two steel buttons: one button attaches to the base of the first metacarpal and the other button attaches to the second metacarpal. This arrangement suspends the thumb ray and effectively prevents subsidence into the CMC space [15].

The authors of this case report have extensive experience with the suture-button suspensionplasty using the Mini TightRope CMC technique (Arthrex). Using the principles of this technique, we present a novel usage of the suture-button suspensionplasty to treat a patient with persistent thumb metacarpal dislocation at the CMC joint. This method, which is in contrast to the described method of volar beak ligament reconstruction using suture-button suspensionplasty, allows for reconstruction of the dorsal ulnar ligament and thereby treats the metacarpal dislocation.

## Materials/methods

### Patient for volar beak ligament reconstruction (traditional)

The patient is a 31-year-old female with a history of right-sided thumb and wrist pain. The patient had attempted conservative measures to treat her pain, including night splinting and non-steroidal anti inflammatory drug (NSAID) treatment, without alleviation of her symptoms. On evaluation, she was noted to have tenderness to palpation at the thumb CMC joint. Although the grind test was negative, joint manipulation demonstrated some laxity with reproducible symptoms. X-rays demonstrated no arthritic changes, but did show some degree of joint laxity at the thumb metacarpal–CMC interface, indicating some ligamentous laxity of the volar beak ligament. The patient underwent operative reconstruction of the volar beak ligament.

### Operative technique for volar beak ligament reconstruction (traditional)

The operative technique for volar beak ligament reconstruction in the scenario of subluxation is the same as described in the literature for first CMC arthritis using suture button suspension, without the trapeziectomy [15].

### Patient for dorsal ulnar ligament reconstruction (novel)

**Figure 1.**
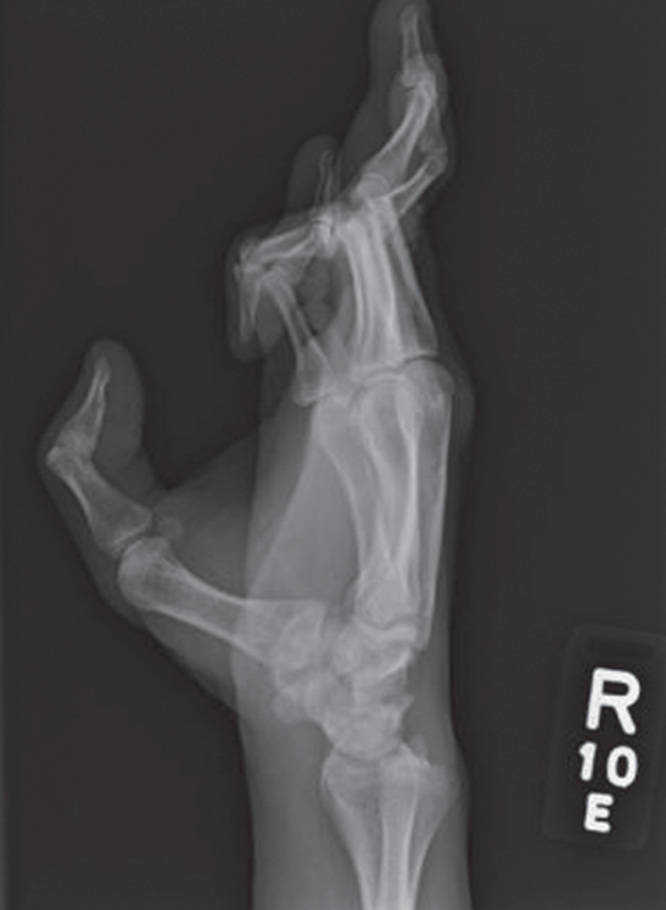
**Lateral preoperative x-rays. The patient demonstrates Z deformity and dislocation of first metacarpal. No arthritic changes are evident over the joint surfaces**

The patient is a 28-year-old male with a history of Klippel–Feil syndrome and joint laxity. The patient had previously experienced proximal first metacarpal dislocation, likely related to the joint laxity caused by his genetic syndrome. During his previous consultations, the patient demonstrated instability of his volar oblique ligament and subsequently underwent an attempt at stabilization of the thumb CMC joint through a ligamentous reconstruction and tendon interposition using a split flexor carpi radialis tendon (without trapeziectomy) approximately 5 years prior. The patient presented to the senior author with similar complaints as he had prior to his original surgery. Subjectively, the patient stated the base of his thumb would dislocate ‘outwards’. The patient had pain and difficulty with movement, and subsequently, had limited range of motion. On physical examination, the patient demonstrated a ‘Z’ deformity of his thumb. The patient had pain upon passive and active range of motion. Grind test was equivocal. Upon ROM of the thumb, the patient’s metacarpal base would subluxate both dorsally and radially, causing the patient significant discomfort. X rays were obtained, confirming the above-mentioned physical examination findings. X-rays demonstrated no evidence of osteophytes or arthritic changes (See Figure 1). The patient was booked for intervention to stabilize the joint.

### Operative technique for dorsal ulnar ligament reconstruction (novel)

**Figures 2.**
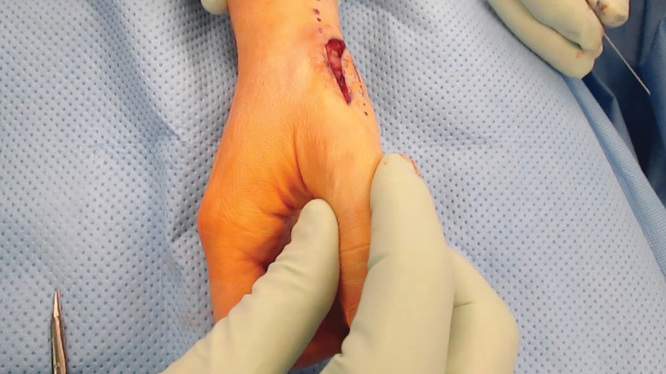
**Intraoperative photograph demonstrating pattern of dislocation.**

**Figures 3.**
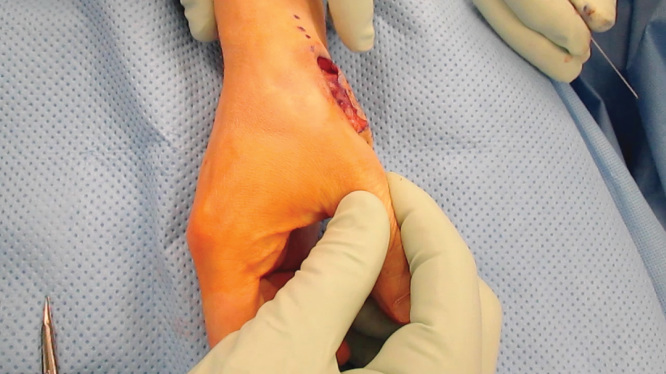
**Intraoperative photograph demonstrating pattern of dislocation.**

**Figure 4.**
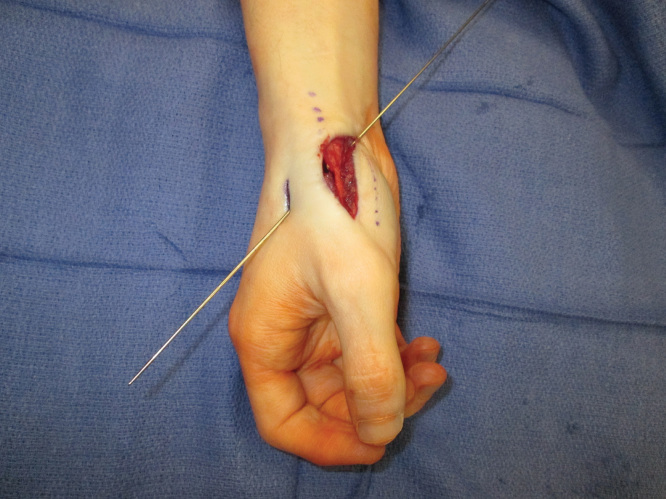
**Intraoperative photograph demonstrating 0.045 K-wire driven across the trapezium into the base of the thumb metacarpal from the dorsal and ulnar side of the metacarpal into the volar and radial side of the trapezium.**

**Figure 5.**
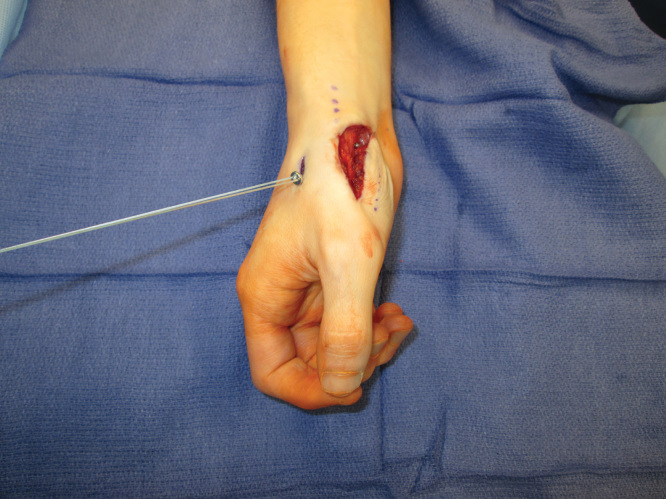
**Intraoperative photograph demonstrating tightening of the Mini TightRope**

**Figure 6.**
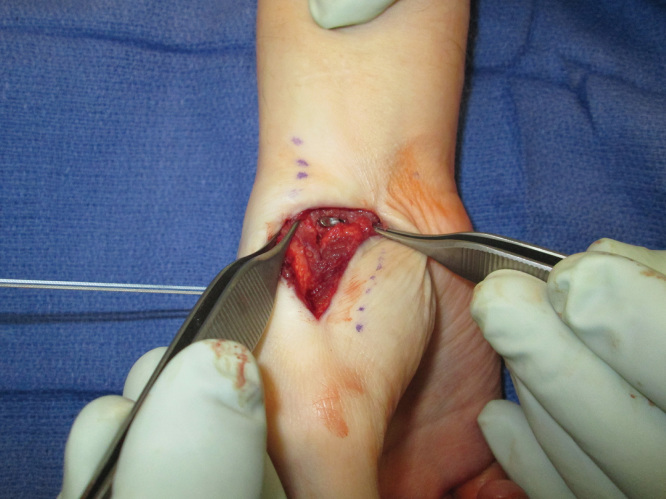
**Intraoperative photograph demonstrating the final placement of fully deployed Mini TightRope.**

**Figure 7.**
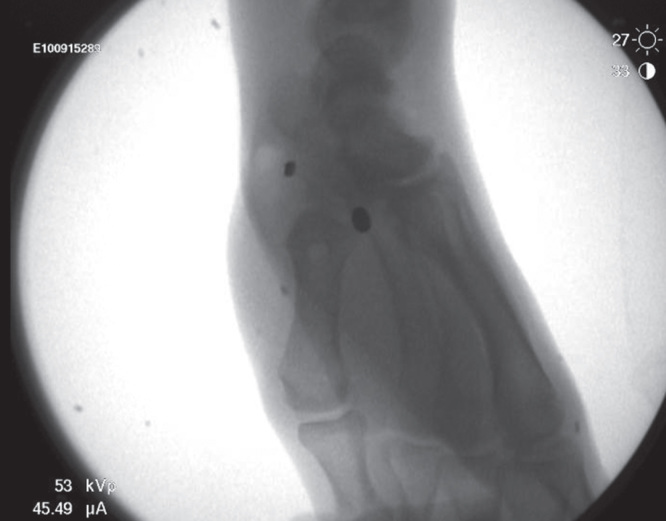
**Fluoroscopic images confirming adequacy of reduction and appropriate placement of suture buttons.**

As mentioned, the patient had previous ligamentous reconstruction performed using a split Flexor Carpi Radialias (FCR). Therefore, surgical options for stabilization were limited. Trapeziectomy was thought to be unnecessary because pre-operative x-rays demonstrated no evidence of arthritic changes to the joint. The decision was then made to reconstruct the dorsal ulnar ligament using the Mini TightRope CMC stabilization technique in a novel fashion – instead of suspending the first metacarpal to the second metacarpal as described above, the first metacarpal was stabilized by suspending it to the trapezium. Ligamentous instability and dislocation is demonstrated (See Figures 2 and 3). Dissection was carried down to the first extensor compartment, which was then released proximally. The dorsal and ulnar side of the CMC joint was identified. It was noted that the first metacarpal was subluxating dorsally in this area. Dissection then proceeded volarly and radially around the trapezium, and the entry point on the trapezium was identified using a 25-gauge needle and fluoroscopy. The previously reconstructed beak ligament using split FCR appeared stable. The senior surgeon planned to reconstruct the dorsal ulnar ligament by placing a Mini TightRope from the dorsal and ulnar side of the metacarpal into the volar and radial side of the trapezium. Once the trajectory was planned, a 0.045 K-wire was then driven across the trapezium into the base of the thumb metacarpal (See Figure 4). Next, the drill from the Mini TightRope set was used to overdrill the hole between the trapezium and the base of the thumb metacarpal over the K-wire that had previously been inserted. The K-wire was then replaced with the suture-passing device, and the Mini TightRope was passed. The button on the volar side was then deployed and the Mini TightRope was tightened (See Figure 5). There was excellent stability of the CMC joint with no dorsal or ulnar dislocation. The Mini TightRope was tightened and tied (See Figure 6), and mini C-arm images were used to confirm proper alignment with lack of dislocation. The knot was buried dorsally and then the skin incisions were closed. Intraoperative fluoroscopy was used to confirm the adequacy of placement of the Mini TightRope (See Figure 7). The patient was placed in a thumb spica splint, and discharged home.

### Postoperative

**Figures 8.**
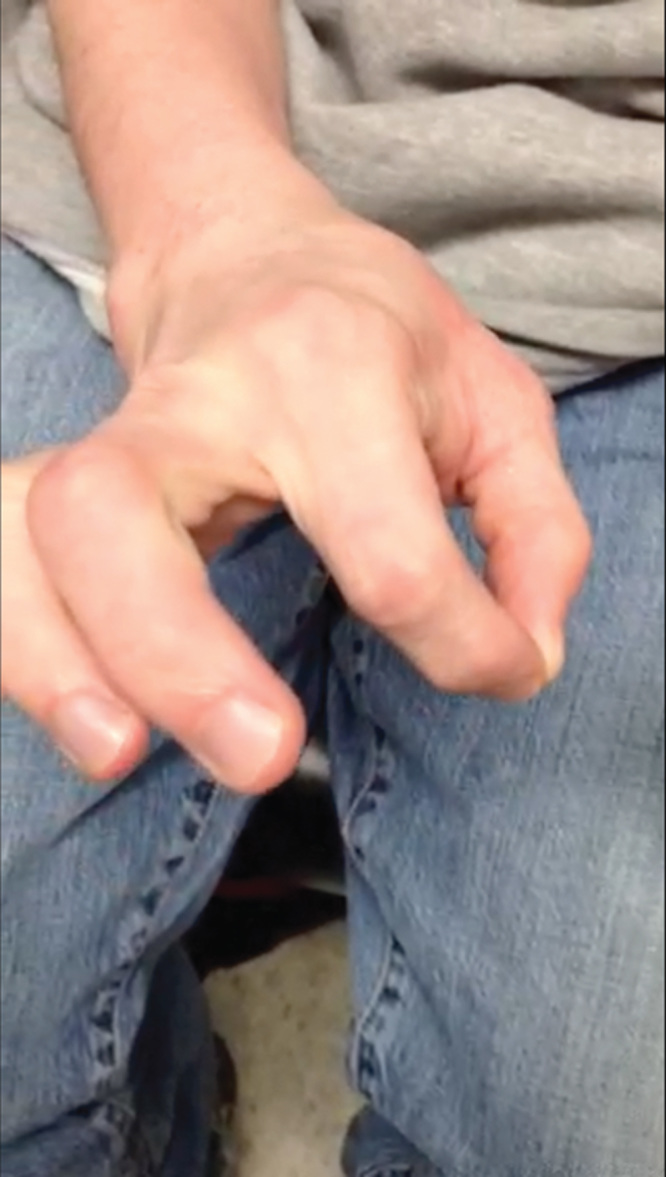
**Demonstration of appropriate functionality of hand and thumb–CMC interface. Patient demonstrates no residual dislocation or dorsal ulnar ligament laxity. Patient has appropriate movements associated with thumb–CMC joint, and has appropriate pinch and grip strength.**

**Figures 9.**
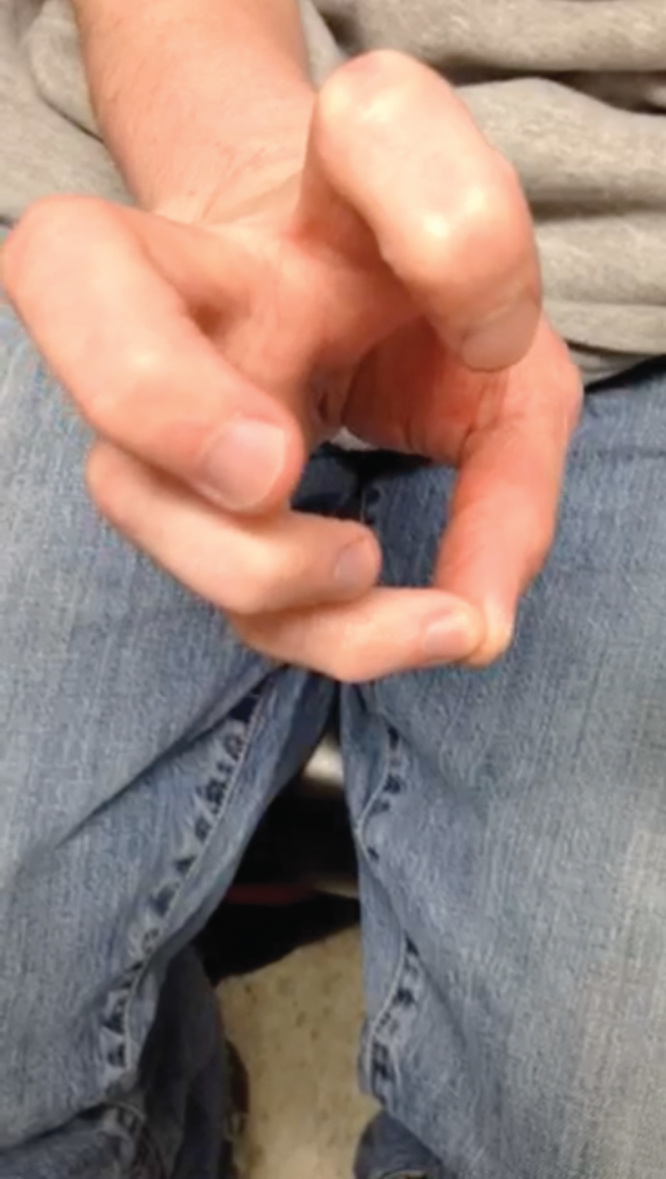
**Demonstration of appropriate functionality of hand and thumb–CMC interface. Patient demonstrates no residual dislocation or dorsal ulnar ligament laxity. Patient has appropriate movements associated with thumb–CMC joint, and has appropriate pinch and grip strength.**

The patient was seen in follow-up clinic. The splint was removed, and sutures were removed at 14 days postoperatively. The patient started hand therapy with significant improvement in function. At 6 weeks follow-up, the patient demonstrated continued stability of the thumb CMC joint without dorsal or radial dislocation, and demonstrated strong pinch and grip functionality (see Figures 8 and 9). He has been seen at 9 months follow-up since the procedure and has continued stability at the CMC joint, without pain.

## Discussion

Large loads are imparted to the trapezium. As investigated in previous reports, the trapezium would be inherently unstable due to its anatomic location without strong supporting ligaments [2]. The attenuation of the strong supporting ligamentous structures leads to instability of the basilar joint and subsequent arthritis.

The authors of this case report present a novel usage of the suture-button suspensionplasty to treat a patient with proximal thumb metacarpal dislocation at the trapezial–metacarpal interface. Instead of suspending the thumb metacarpal to the index metacarpal, as is done for traditional volar beak ligament reconstruction, the authors have suspended the first metacarpal to the trapezium. This method allowed reconstruction of the dorsal ulnar ligament using the Mini TightRope (Arthrex), thereby allowing stabilization of the joint by preventing dorsal and radial dislocation of the metacarpal. Joint arthrodesis could have been considered in this patient. However, by performing a full arthrodesis, the patient would use significant functionality of the thumb CMC joint as the adduction/abduction arc and flexion/extension arc are significantly reduced. Furthermore, joint arthrodesis has a high rate of delayed union or malunion [16]. This would be detrimental to a young patient whose main problem is joint laxity causing pain rather than pain associated with arthritic changes. Our patient has avoided joint arthrodesis, and as demonstrated in the above images, still has appropriate motion at this joint.

It is unclear whether there will be post-operative complications with the usage of the suture-button suspension in the described fashion. To date, there has been no indication of arthritis or joint erosion. Long-term follow-up will be necessary to evaluate for arthritis or failure of suspension.

As stated by Pagalidis *et* *al*, in first CMC arthritis, clinical and radiological evidence of radial and dorsal or dorso-proximal subluxation of the base of the first metacarpal is frequently demonstrated. They state that the key ligamentous structure in maintaining stability at this joint should therefore be located on the ulnar and volar aspect of the joint exerting a constraining effect on the base of the thumb against such displacement [17]. Intuitively then, the dorsal ulnar ligament would therefore be important to first CMC joint stability. However, due to the importance of the beak ligament in the integrity of the joint, the reconstruction of this ligament alone may not provide sufficient stability of the joint.

## Conclusion

This technique demonstrates the benefits associated with ligamentous reconstruction, while also avoiding the problems associated with joint arthrodesis. This novel technique allows hand surgeons the ability to stabilize the first metacarpal to the trapezium when other options are either unavailable or less desirable.
